# Serum calcium–phosphorus product for predicting the risk of osteoporotic vertebral compression fractures in elderly patients: a retrospective observational study

**DOI:** 10.1186/s13018-022-02953-5

**Published:** 2022-01-29

**Authors:** Pu Ying, Mingjia Gu, Xiaowei Jiang, Yue Xu, Lu Tong, Yi Xue, Qiang Wang, Zhihui Huang, Wenge Ding, Xiaoyu Dai

**Affiliations:** 1 Department of Orthopedics, Changshu Hospital Affiliated to Nanjing University of Chinese Medicine, No. 6 Huanghe Road, Changshu, 215500 China; 2 Department of Nephrology, Changshu Hospital Affiliated to Nanjing University of Chinese Medicine, No. 6 Huanghe Road, Changshu, 215500 China; 3 Department of Orthopedics, The Third Affiliated Hospital of Soochow University, No. 185 Juqian Road, Changzhou, China

**Keywords:** Serum calcium–phosphorus product, Corrected calcium–phosphorus product, Osteoporotic vertebral compression fracture

## Abstract

**Background:**

This study retrospectively analyzed and evaluated the potential correlations of serum calcium, serum phosphorus, and calcium-phosphorus product (Ca–P product) with the incidence of osteoporotic vertebral compression fractures (OVCFs), with the aim of exploring whether the Ca–P product can be used as a serological indicator to predict the risk of OVCFs.

**Methods:**

This study randomly enrolled 400 elderly patients in our hospital with OVCFs and 400 patients with hip and knee arthroplasty due to femoral head necrosis or osteoarthritis from August 2013 to April 2021. Age, sex, past medical history, and admission biochemical indicators, including albumin, blood urea nitrogen, serum creatinine, serum calcium and serum phosphorus, were collected for statistical analysis.

**Results:**

Albumin, serum calcium, serum phosphorus, Ca–P product, corrected serum calcium and corrected Ca–P product were lower in the OVCF group than in the non-OVCF group (*P* < 0.05). Multivariate logistic regression analysis showed that low values of serum calcium, serum phosphorus, Ca–P product, corrected blood calcium, and corrected Ca–P product can all be risk factors for OVCF. The ROC curve showed that the Ca–P product and corrected Ca–P product were effective in predicting the risk of OVCFs. The predictive value of the Ca–P product was the best; the cutoff point was 29.88, the sensitivity was 0.72 and the specificity was 0.62. The cutoff point of the corrected Ca–P product was 30.50, the sensitivity was 0.74, and the specificity was 0.62.

**Conclusion:**

The Ca–P product and corrected Ca–P product can be used as serological indicators to predict the risk of OVCFs in elderly individuals. Early clinical interventions targeting this risk factor can further reduce the risk of OVCFs. Also, timely and regular testing of the serum calcium and phosphorus level is recommended and encouraged for this group of people.

## Introduction

Osteoporotic vertebral compression fracture (OVCF) is a common clinical fracture type. As the most common osteoporotic fracture, the incidence of OVCFs in the elderly is very high [1, 2]. Studies have shown that the incidence of OVCFs may be as high as 30.0–50.0% in the global population over 50 years of age [3]. Osteoporosis is the most direct cause of OVCFs and can lead to the destruction of bone microstructure, degradation of bone quality and reduction of bone mineral density.

The clinical prediction of osteoporosis risk mainly relies on bone mineral density detection and bone transformation markers [4, 5], but there is still a lack of relatively effective serological indicators that can predict the risk of OVCFs. In this regard, it is worth mention that the circumstances and organization of the existing health care system may limit us to focus on the value of the serum calcium and phosphorus level variables in the prediction of OVCFs. Actually, previous studies have shown that serum calcium, serum phosphorus, and calcium and phosphorus metabolism are closely related to osteoporosis [6, 7], but their specific correlations with the occurrence of OVCFs have not been reported and need further confirmation.

Based on the simplicity of the clinical detection of serum calcium and serum phosphorus, this study retrospectively analyzed and evaluated the potential correlations of serum calcium, serum phosphorus, and calcium-phosphorus product (Ca–P product) with the incidence of OVCFs, aiming to explore whether the Ca–P product can be used as a serological indicator to predict the risk of OVCFs.

## Methods

### Inclusion and exclusion criteria

This study included 400 patients with OVCFs seen in our department from August 2013 to April 2021 in the observation group and 400 patients with hip and knee arthroplasty due to end-stage femoral head necrosis or osteoarthritis during the same period in the control group. The inclusion criteria were as follows: (1) Age > 60 years old with no obvious trauma or only mild trauma history; (2) OVCF diagnosed by medical history, physical examination, imaging examination and/or bone mineral density examination; (3) Course of disease < 3 weeks; (4) Normal thyroid and parathyroid functions and no history of thyroid or parathyroid diseases, surgical history or family history; and (5) Normal gastrointestinal tract, liver and kidney functions and no major systemic disease. The exclusion criteria were as follows: (1) Fractures caused by major violence; (2) Malignant tumor or skeletal system with primary or metastatic tumor lesions; (3) Gastrointestinal, hepatic, renal insufficiency or major diseases; (4) Bone metabolism-related endocrine diseases (parathyroid disease, gonadal disease, adrenal disease and thyroid disease), rheumatoid arthritis and other immune system diseases; and (5) Long-term administration of glucocorticoids or other drugs affecting bone metabolism. This study was approved by the ethics committee of Changshu Hospital Affiliated with Nanjing University of Chinese Medicine, and all patients signed informed consent forms.

### Observation indices

Clinical data were collected on admission and included age, sex, and past medical history of hypertension and diabetes. Additionally, the following initial biochemical indices were collected: albumin, urea nitrogen, serum creatinine, serum calcium and serum phosphorus. Blood samples were collected on admission. Corrected serum calcium was calculated according to serum calcium. The correction formulas were as follows:

1. Corrected serum calcium (mmol/L) = serum calcium (mmol/L) + (40 − serum albumin) × 0.025 (mmol/L) [8].
2. Serum calcium: 1 mmol/L = 4 mg/dl, serum phosphorus: 1 mmol/L = 3.1 mg/dl.
3. Ca–P product = (4 × serum calcium) × (3.1 × serum phosphorus).
4. Corrected Ca–P product = (4 × corrected blood calcium) × (3.1 × serum phosphorus).

### Statistical analysis

All data were analyzed by SPSS 22.0 (SPSS, Chicago), and the chi-square test was used for comparisons between groups of classified data. Independent sample t-tests were used for comparisons between groups of measurement data with a normal distribution, and Mann–Whitney U tests were used for comparisons between groups of measurement data without a normal distribution. Logistic regression was used to analyze the abilities of serum calcium, serum phosphorus, Ca–P product, corrected serum calcium, corrected Ca–P product and other indicators to predict OVCF. Finally, the receiver operating characteristic curve (ROC) curve was drawn. According to the area under the curve (AUC), the model with good reliability in the prediction model was evaluated, and the cutoff point of risk factors was determined. *P* < 0.05 indicated that the difference was statistically significant.

## Results

### Patient characteristics

In Table 1, the average age of the OVCF group and non-OVCF group was 71.50 ± 6.41 years old and 72.43 ± 8.04 years old, respectively, and the difference was not statistically significant (*P* > 0.05). There was no significant difference in sex, hypertension, diabetes, urea nitrogen or creatinine between the two groups (*P* > 0.05). However, the albumin, serum calcium and serum phosphorus in the observation group were lower than those in the control group, and the difference was statistically significant (*P* < 0.05). After calculating the corrected values using the correction formulas, the Ca–P product, corrected blood calcium and corrected Ca–P product in the OVCF group were lower than those in the non-OVCF group, and the difference was statistically significant (*P* < 0.05).

**Table 1:** Comparison of clinical and corrected data between the observation and control groups

Clinical characteristics	OVCF	Non-OVCF	*t/χ*^2^	*P* value
Patients, no	400	400		
Mean age, years	71.50 ± 6.41	72.43 ± 8.04	*t* = −1.286	0.199
Sex, no. (male: female)	78: 322	102: 298	*χ*^*2*^ = 2.065	0.151
Hypertension, no	94	114	*χ*^*2*^ = 1.672	0.196
Diabetes mellitus, no	56	68	*χ*^*2*^ = 0.687	0.407
Albumin, g/L	37.70 ± 3.47	38.35 ± 2.74	*t* = −2.088	0.037
Blood urea nitrogen, mmol/L	5.48 ± 1.45	5.68 ± 1.33	*t* = −1.455	0.146
Serum creatinine, μmol/L	61.83 ± 12.59	60.70 ± 12.06	*t* = 0.917	0.360
Serum calcium, mmol/L	2.22 ± 0.12	2.26 ± 0.13	*t* = −3.667	< 0.001
Serum phosphorus, mmol/L	1.03 ± 0.13	1.13 ± 0.12	*t* = −7.988	< 0.001
Ca–P product	28.40 ± 4.09	31.87 ± 3.84	*t* = −8.482	< 0.001
Corrected serum calcium, mmol/L	2.27 ± 0.09	2.30 ± 0.13	*t* = −2.590	0.010
Corrected Ca–P product	29.14 ± 3.97	32.36 ± 3.84	*t* = −8.294	< 0.001

### Multivariate analysis

In the multivariate logistic regression analysis, low serum calcium (OR = 0.051; 95% CI = 0.010–0.255; *P* < 0.001), low serum phosphorus (OR = 0.002; 95% CI = 0.000–0.011; *P* < 0.001), low Ca–P product (OR = 0.073; 95% CI = 0.036–0.148; *P* < 0.001), low corrected serum calcium (OR = 0.112; 95% CI = 0.019–0.649; *P* = 0.015), and low corrected Ca–P product (OR = 0.072; 95% CI = 0.035–0.149; *P* < 0.001) were significant risk factors for OVCF after sex and age correction (Table 2).

**Table 2:** Multivariate logistic regression analysis of risk factors for OVCFs

Risk factors	OR	95% CI	*P* value
Serum calcium	0.051	0.010–0.255	< 0.001
Serum phosphorus	0.002	0.000–0.011	< 0.001
Ca–P product	0.073	0.036–0.148	< 0.001
Corrected serum calcium	0.112	0.019–0.649	0.015
Corrected Ca–P product	0.072	0.035–0.149	< 0.001

## ROC analysis

**Fig. 1:**
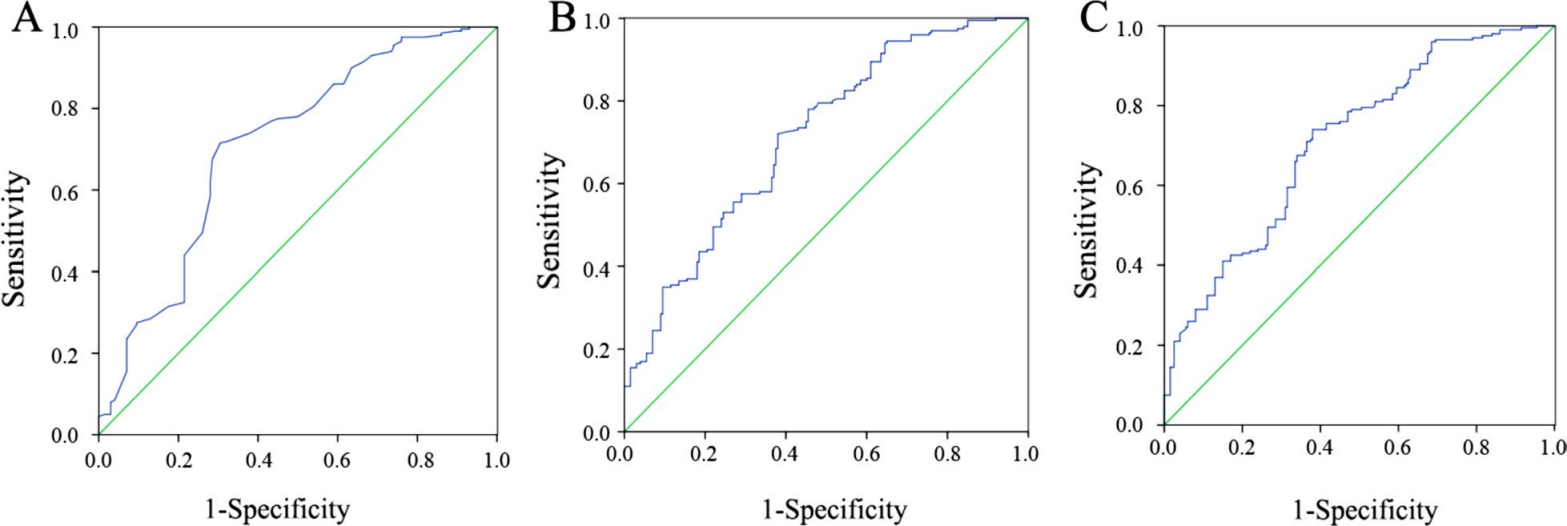
ROC curve of serum phosphorus, Ca–P product and corrected Ca–P product in predicting OVCFs. **A** Serum phosphorus; **B** Ca–P product; **C** Corrected Ca–P product

In the ROC analysis, we calculated the area under the curve (AUC) of different risk factors and found that serum phosphorus, Ca–P product and corrected Ca–P product were predictive factors for OVCFs. The AUCs of serum phosphorus, the Ca–P product and the corrected Ca–P product were 0.709, 0.714, and 0.712, respectively (Table 3). The ROC curves of the 3 risk factors are shown in Fig. 1. The optimum cutoff point for the Ca–P product was 29.88, with a sensitivity of 0.72 and specificity of 0.62; the optimum cutoff point for the corrected Ca–P product was 30.50, with a sensitivity of 0.74 and specificity of 0.62; and the optimum cutoff point for serum phosphorus was 1.085, with a sensitivity of 0.715 and specificity of 0.695.

**Table 3:** The ROC Results of Risk Factors to Predict OVCF

Risk factors	AUC	SE	95% CI	*P* value
Serum calcium	0.602	0.029	0.546–0.658	< 0.001
Serum phosphorus	0.709	0.026	0.659–0.760	< 0.001
Ca–P product	0.714	0.025	0.664–0.764	< 0.001
Corrected serum calcium	0.580	0.029	0.523–0.636	0.006
Corrected Ca–P product	0.712	0.025	0.662–0.762	< 0.001

## Discussion

With the growth of the elderly population, the number of fractures caused by osteoporosis is expected to increase annually. The incidence and mortality of OVCFs in the elderly population are rapidly increasing [9, 10]. Therefore, it is particularly important to screen out individuals at high risk for OVCFs in the elderly population and carry out early intervention. Existing studies have shown that sex, age, bone mineral density, and bone transformation markers are closely related to the occurrence of OVCFs [5, 11]. Bone mineral density examination is mainly performed by dual-energy X-ray absorptiometry. The inspection equipment is relatively expensive, the execution is relatively complex, and there are shortcomings such as X-ray radiation exposure. It has been reported that the accuracy of using bone mineral density alone to predict osteoporotic fractures remains to be further improved [12]. Bone transformation markers, including bone resorption markers and bone formation markers, such as N-terminal propeptide of type I procollagen (PINP), C-telopeptide of type I collagen (CTX-I) and osteocalcin, have many limitations in clinical application [13] and still cannot be used as diagnostic markers for individualized diagnosis and treatment of osteoporotic fractures.

In this study, we retrospectively investigated the serum Ca–P product and its correlation with OVCFs. The results showed that the serum calcium, serum phosphorus, corrected serum calcium, Ca–P product and corrected Ca–P product in patients with OVCFs were lower than those of individuals in the same age group with relatively healthy bones. Serum calcium and phosphorus metabolism has been proven to be an important part of bone metabolism. The main factors that maintain blood calcium and phosphorus are 1,25-dihydroxyvitamin D3 (1,25(OH)2 D3), parathyroid hormone (PTH), and fibroblast growth Factor 23 (FGF23) [14–16]. Blood calcium and phosphorus in the human body maintain a relative balance and play an important role in the differentiation of osteoblasts and osteoclasts [17]. The concentrations of calcium and phosphorus in human blood are closely related. Low calcium levels may lead to an increase in parathyroid hormone (PTH) and then promote bone to release calcium ions into the blood, increase renal phosphorus excretion, and maintain serum calcium and phosphorus homeostasis [18, 19]. The product of blood calcium and phosphorus is closely related to osteogenesis and osteolysis. When the product of calcium and phosphorus is high, calcium and phosphorus are deposited in bone tissue. When the product of calcium and phosphorus is low, the calcification of bone is inhibited, and osteogenesis is affected.

To the best of our knowledge, the Ca–P product has not been used as a predictor of OVCFs in previous studies, and our study concluded that the Ca–P product can be used as a new and useful predictor of OVCFs. We further quantitatively analyzed the best indicators for predicting risk efficacy and found that when the Ca–P product of the elderly individuals is less than 29.88 or the corrected Ca–P product was less than 30.50, we should pay close attention to the risk of OVCFs and carry out standardized and scientific anti-osteoporosis interventions as soon as possible to reduce the incidence of OVCFs and the resulting decline in the quality of life and even death risk in the elderly population.

In this study, patients with osteonecrosis of the femoral head or osteoarthritis undergoing knee and hip replacement were randomly selected as the control group. The average age was similar, which potentially reduced the impact of age on bone metabolism and increased the reliability of the results to some extent. However, considering that the sample size of this study was still small, the ratio of males to females was quite different, and there was a lack of supporting multicenter and large sample data. Additionally, there were many uncontrolled biases related to calcium and phosphorus levels in blood, such as magnesium concentration, vitamin D levels, type of diet, and drugs taken by the patient, all of which were not mentioned. Moreover, we failed to compare the serum Ca–P product with bone mineral density and bone transformation markers to obtain the predictive value and effectiveness of the indicators for the occurrence of OVCFs. In addition, the prediction sensitivity and specificity of the Ca–P product and corrected Ca–P product for OVCFs in this study were not very high, and the prediction accuracy needed to be improved.

## Conclusions

This study preliminarily confirmed that the serum Ca–P product and corrected Ca–P product could be used as independent serological indicators to predict the risk of osteoporotic vertebral compression fractures in elderly individuals. Early effective clinical interventions may reduce the risk of osteoporotic fractures. Additionally, our findings imply a breakthrough point for subsequent multicenter studies and the management, diagnosis, and treatment of osteoporosis-related diseases in grassroots hospitals in China.

## Data Availability

The datasets used and analyzed during the current study are available from the corresponding author on reasonable request.

## Abbreviations

OVCF: Osteoporotic vertebral compression fracture
ROC: Receiver operating characteristic curve
AUC: Area under the curve
Ca–P product: Calcium–phosphorus product
